# Complete Genome Sequence of *Bordetella pertussis* Strain VA-190 Isolated from a Vaccinated 10-Year-Old Patient with Whooping Cough

**DOI:** 10.1128/genomeA.00972-16

**Published:** 2016-09-15

**Authors:** Joshua C. Eby, Lauren Turner, Bryan Nguyen, June Kang, Carly Neville, Louise Temple

**Affiliations:** a University of Virginia Health Systems, Charlottesville, Virginia, USA; b Virginia Division of Consolidated Laboratory Services, Richmond, Virginia, USA; c James Madison University, Harrisonburg, Virginia, USA

## Abstract

The number of cases of pertussis has increased in the United States despite vaccination. We present the genome of an isolate of *Bordetella pertussis* from a vaccinated patient from Virginia. The genome was sequenced by long-read methodology and compared to that of a clinical isolate used for laboratory studies, D420.

## GENOME ANNOUNCEMENT

Pertussis, or whooping cough, is the respiratory illness caused by infection with *Bordetella pertussis*. In infants, pertussis can result in respiratory distress and death. After introduction of whole cell pertussis vaccines (wP) in the United States in 1942, reported cases of pertussis decreased from >200,000 per year to 1,010 in 1976 (1). Since that time there has been an increase in cases, with a recent high of >48,000 in 2012. The greatest increase correlated with a change from wP to acellular pertussis vaccine (aP) in the 1990s, and rapidly waning immunity to aP, poor efficacy of aP, and genetic adaptation of *B. pertussis* to antigens in aP may all contribute to the observed incidence. Genetic analysis of clinical isolates of *B. pertussis* can be used to understand the contribution of genetic adaptation to pertussis reemergence. The clinical isolate presented here was obtained in Virginia in 2012 from a 10-year-old female patient who acquired pertussis despite immunization with aP.

The isolate was obtained from the Virginia Division of Consolidated Laboratory Services. SMRTbell DNA libraries were constructed according to Pacific Biosciences (Pacbio) standard 20-kb library. Libraries were size-selected using BluePippin (Sage Science) starting at 7 kb with an average library size of 20 to 22 kb. Sequencing was performed on PacBio RSII with one SMRT cell, yielding 91,332 reads, *N*_50_ read length of 8,732, and 100× coverage. The 4.1-Mb genome was assembled *de novo* using Celera and HGAP.3. The genome was annotated using RAST (2). To identify single nucleotide polymorphisms (SNPs), Mauve (3) was used to map genome of isolate VA-190 against the genome of strain D420 (4).

VA-190 has a 67.7% G+C content and 4,145 predicted genes. There are 3,938 orthologs and 61 SNPs compared to D420. The SNPs are located mainly in IS481 elements (34), intergenic regions (6), and hypothetical proteins (21). VA-190 has pertussis toxin (*ptxP3)* and fimbrae (*fim3-2)* alleles similar to those in recent reports (5). In strain VA-190, the pertactin gene (*prn*) is truncated by a stop codon at base 3,324,137 in the genome. A deletion of 228 bases occurs at this point, producing a stop codon in the *prn* reading frame. *prn* is changed in 30 to 85% isolates collected from 1997 to the present in several countries (6–10). These reported defects are caused by IS481 insertions, deletions, and SNPs in locations across the length of the gene.

VA-190 and D420 share genomic synteny with the exception of two inverted regions between coordinates 2,265,549 and 2,689,693 in VA-190. The only other exception is an intergenic region in VA-190 (coordinates 2,820,180 to 2,820,630) that does not share similarity with D420. Ends of inverted regions occur at tRNA genes, rRNA genes or in intergenic regions, thus are likely not to change protein content.

This study suggests that sequencing by PacBio technology is an effective way to characterize the genome of an organism that exhibits frequent insertions, deletions, and rearrangements (11, 12). VA-190 adds to the growing public data set of *B. pertussis* genomes sequenced by long-read methodology, and available for understanding pathogenesis and genetic change associated with pertussis vaccines rearrangements (11, 13).

### Accession number(s).

The complete genome of VA-190 is deposited in GenBank under accession number CP015761.
